# Effect of increased protein intake on renal acid load and renal hemodynamic responses

**DOI:** 10.14814/phy2.12687

**Published:** 2016-03-20

**Authors:** Karianna F. M. Teunissen‐Beekman, Janneke Dopheide, Johanna M. Geleijnse, Stephan J. L. Bakker, Elizabeth J. Brink, Peter W. de Leeuw, Marleen A. van Baak

**Affiliations:** ^1^ Top Institute Food and NutritionWageningenThe Netherlands; ^2^ Department of Human BiologyNUTRIM School for Nutrition, Toxicology and MetabolismMaastricht UniversityMaastrichtThe Netherlands; ^3^ Division of Human NutritionWageningen UniversityWageningenThe Netherlands; ^4^ Department of MedicineUniversity Medical Center Groningen and University of GroningenGroningenThe Netherlands; ^5^ Department of MedicineMaastricht University Medical Center and Cardiovascular Research Institute Maastricht (CARIM)MaastrichtThe Netherlands

**Keywords:** Acid load, carbohydrate, glomerular filtration rate, kidney, protein

## Abstract

Increased protein intake versus maltodextrin intake for 4 weeks lowers blood pressure. Concerns exist that high‐protein diets reduce renal function. Effects of acute and 4‐week protein intake versus maltodextrin intake on renal acid load, glomerular filtration rate and related parameters were compared in this study. Seventy‐nine overweight individuals with untreated elevated blood pressure and normal kidney function were randomized to consume a mix of protein isolates (60 g/day) or maltodextrin (60 g/day) for 4 weeks in energy balance. Twenty‐four‐hour urinary potential renal acid load (uPRAL) was compared between groups. A subgroup (maltodextrin *N* = 27, protein mix *N* = 25) participated in extra test days investigating fasting levels and postprandial effects of meals supplemented with a moderate protein‐ or maltodextrin‐load on glomerular filtration rate, effective renal plasma flow, plasma renin, aldosterone, pH, and bicarbonate. uPRAL was significantly higher in the protein group after 4 weeks (*P ≤ *0.001). Postprandial filtration fraction decreased further after the protein‐supplemented breakfast than after the maltodextrin‐supplemented breakfast after 4 weeks of supplementation (*P ≤ *0.001). Fasting and postprandial levels of glomerular filtration rate, effective renal plasma flow, renin, aldosterone, angiotensin‐converting enzyme, pH and bicarbonate did not differ between groups. In conclusion, 4 weeks on an increased protein diet (25% of energy intake) increased renal acid load, but did not affect renal function. Postprandial changes, except for filtration fraction, also did not differ between groups. These data suggest that a moderate increase in protein intake by consumption of a protein mix for 4 weeks causes no (undesirable) effects on kidney function in overweight and obese individuals with normal kidney function.

## Introduction

Increased protein diets are often advised to overweight and obese persons because these diets may reduce adiposity (Santesso et al. 2012). A blood pressure lowering effect of 4 weeks of increased protein consumption (3 × 20 g/day) compared with 4 weeks of maltodextrin consumption (3 × 20 g/day) in overweight individuals with mild blood pressure elevation was reported previously (Teunissen‐Beekman et al. 2012). Because overweight and obese persons often have elevated blood pressure (Kang 2013), this may be an extra benefit from consuming a high‐protein diet for this group. However, small beneficial health effects of high‐protein diets need to be weighed against potential harms (Santesso et al. 2012), like gradual loss of kidney function (Martin et al. 2005). Among the mechanisms held responsible for the effects of dietary protein on the kidney are hyperfiltration (Palatini 2012) and increased renal acid load (van den Berg et al. 2011; Goraya and Wesson 2012). Previous food‐based studies have shown that diets with increased protein content increase glomerular filtration rate (GFR) acutely (Viberti et al. 1987; Chan et al. 1988; Simon et al. 1998) and after chronic consumption (Skov et al. 1999; Frank et al. 2009; Juraschek et al. 2013) in individuals with normal kidney function. We investigated GFR and acid load in response to a ≈ 10% increase in energy intake from protein in overweight individuals with mild blood pressure elevation and normal kidney function. Chronic and postprandial responses of renal acid load, renal hemodynamics, renal function parameters, and the renin‐angiotensin‐aldosterone system were compared between protein‐ and maltodextrin‐supplemented diets (Teunissen‐Beekman et al. 2012, 2013). Supplements consisting of protein isolates (a mix of milk, egg‐white, soy and pea protein isolates) were chosen and compared to maltodextrin to minimize for confounding effects of other nutrients present in food. In contrast to previous studies (Viberti et al. 1987; Chan et al. 1988; Simon et al. 1998), only a moderate difference in protein intake was tested during the postprandial tests because this difference would be more realistically achieved during a high protein diet with a 10% increase in energy from dietary protein. We hypothesized that the renal acid load and fasting GFR would be higher on the protein‐supplemented diet than on the maltodextrin‐supplemented diet and that the protein‐supplemented breakfast would induce a greater postprandial increase in GFR and effective renal plasma flow (ERPF) than the maltodextrin‐supplemented breakfast both at the start and at the end of the 4‐week diet intervention. Because the kidneys are involved in blood pressure regulation, postprandial changes in renin and aldosterone were also measured. Angiotensin‐converting enzyme (ACE) activity was studied, because (milk)proteins are hypothesized to reduce blood pressure via ACE inhibition (McGregor and Poppitt 2013).

## Material and Methods

This randomized, double blind, parallel group trial on the effects of protein on blood pressure (PROPRES) has been described previously (Teunissen‐Beekman et al. 2012, 2013). All 94 participants followed a standard dietary advice (Energy: 15% protein, 55% carbohydrate, 30% fat) starting 2 weeks before the intervention and ending after the intervention period. During the 4‐week intervention period the groups exchanged 3 × 20 g/day of carbohydrates in their diet with 3 × 20 g/day of a protein mix (20% pea protein, 20% soy protein, 30% egg‐white protein and 30% milk protein) or with 3 × 20 g/day of maltodextrin. As an example, protein intake would be increased from 79 g/day to 139 g/day for a diet of 2000 kcal per day. Twenty‐four‐hour urine was collected before the start (day 0) and at the end of the intervention period. Postprandial renal and humoral responses to protein‐ and maltodextrin‐supplemented meals were compared in a subgroup (*N* = 52) of the PROPRES study on day 1 and after 4 weeks of supplementation. The study adhered to the Declaration of Helsinki was approved by the local medical ethical committee of Maastricht University Medical Centre. All participants gave written informed consent. Inclusion and exclusion criteria and the method of randomization are described elsewhere (Teunissen‐Beekman et al. 2012, 2013).

### Test days

Participants attended test days on day 1 and after 4 weeks of supplementation for measurements of variables in the fasting state and in response to protein‐ or maltodextrin‐supplemented meals. Meals were supplemented with 20 g of the protein mix or 20 g maltodextrin. Renal hemodynamic responses were measured at 30, 60, 90, 120, 180, and 240 min after breakfast. Postprandial changes in active renin, ACE, and aldosterone were also measured during 4 h after breakfast and were continued for 8 more hours at 300, 360, 420, 480, 540, 600, 660, and 720 min. Lunch and dinner were consumed after 4 and 8 hours. Further details on measurements and on the composition of the meals and supplements can be found elsewhere (Teunissen‐Beekman et al. 2012, 2013).

### Renal hemodynamic responses

After participants arrived at the university a catheter was inserted in the left and right antecubital vein and retrogradely in a dorsal vein of the right hand. The right hand was heated in a hot box in order to arterialize the blood in the hand vein. For the determination of GFR and ERPF, inulin (Inutest 25%, Fresenius Kabi, Graz, Austria) and para‐aminohippurate (PAH, MSD, Haarlem, the Netherlands) were continuously infused in the right arm vein and blood was drawn from the left arm vein (van der Zander et al. 2006).

Filtration fraction (FF) was calculated as GFR/ERPF. Effective renal blood flow (ERBF) was calculated as ERPF/(1‐hematocrit). Hematocrit was measured with a microhematocrit reader. Renal vascular resistance (RVR) was calculated from ERBF and MAP as: MAP/ERBF. Measurements of blood pressure can be found elsewhere (Teunissen‐Beekman et al. 2013).

### Renin, ACE, aldosterone

Aldosterone and renin were analyzed in EDTA‐plasma and ACE was analyzed in serum. Analyses were done by MLM medical labs (Mönchengladbach, Germany). ACE activity was determined with the ACE‐color method (Fujirebio, Tokyo, Japan). Renin was measured with the LIAISON Direct renin assay using chemiluminescent immunoassay technology (Diasorin, Saluggia, Italy). Aldosterone was measured with an ELISA (IBL international GMBH, Hamburg, Germany).

### Acid load and urinary excretions

Blood pH and bicarbonate concentration were measured in arterialized blood with a blood gas analyzer (ABL 510, Radiometer Medical A/S, Copenhagen, Denmark).

Renal acid load was determined by calculating the 24‐h urinary potential renal acid load (uPRAL), which reflects urinary net acid excretion (NAE) without its organic anion component (Krupp et al. 2014). UPRAL was calculated as follows:uPRAL (mEq/day) = [Cl (mmol/day) + SO_4_ (mmol/day) * 2 + PO_4_ (mmol/day) * 1.8) − (Na (mmol/day) + K (mmol/day) + Mg (mmol/day) * 2 + Ca (mmol/day) * 2] (Krupp et al. 2014).

Twenty‐four‐hour urinary excretions of electrolytes, sulfate, and phosphate are reported elsewhere (Teunissen‐Beekman et al. 2012). Twenty‐four‐hour urinary excretion of ammonium was measured chromatographically (Alliance HT 2795; Waters, Milford, MA). Ammonium and ammonia play an important role in the elimination of acid from the body (Garibotto et al. 2009). Twenty‐four‐hour urinary pH and titratable acid were measured with an automated titrator (855 Robotic Titrosampler; Metrohm, Herisau, Switzerland). Acid excretion was not measured directly because 24‐h bicarbonate excretion was not available in our study. Protein intake (g/day) was calculated as: [24‐h urinary urea nitrogen excretion (UUN; g/day)] + 0.031 * [body weight (kg)] * 6.25.

### Statistical analyses

Baseline characteristics and fasting values of all variables were tested with an independent samples *t*‐test. Changes in fasting variables, uPRAL, and 24‐h urinary ammonium after 4 weeks of supplementation were compared between groups with an univariate ANCOVA with baseline measurements as a covariate. To control for errors introduced by incorrect 24‐h urine collection, uPRAL and 24‐h urinary ammonium were also analyzed when including only participants who had a change <20% of 24‐h urinary creatinine during the intervention (Stuveling et al. 2004).

Postprandial data on each test day were analyzed by a linear mixed‐model analysis using a random intercept model. Analyses started off with the full model including group, time, fasting measurement, BMI, age and sex. BMI, age. and sex were removed from the model one‐by‐one starting with the least significant covariate to test whether they significantly contributed to the model with a −2 log likelihood test. Significant covariates were kept in the final model. Aldosterone and renin were naturally log‐transformed before analyses because of their non‐normal distributions.

Incremental areas under the curve (iAUCs) were calculated and tested for deviance from zero with a one‐sample *t*‐test to see whether postprandial increases/decreases were significant.

All statistical analyses were performed with SPSS software (version 20; IBM). A *P* value of ≤0.05 was considered significant.

## Results

From the 94 PROPRES participants, 79 were included in the present analyses because uPRAL could not be calculated in some participants due to missing electrolyte concentrations. Baseline characteristics of 43 participants in the maltodextrin group and 36 participants in the protein group included in analyses are shown in Table 1. Baseline characteristics of the total group and the subgroup participating in the postprandial tests were described previously (Teunissen‐Beekman et al. 2012, 2013). Baseline characteristics and 24‐h urinary excretions did not significantly differ between the subgroup participating in the postprandial tests and the total group for (data not shown). Protein intake did not differ between groups at the start of the intervention and was significantly higher in the protein group after 4 weeks of supplementation compared to the maltodextrin group (protein intake (g/day): maltodextrin group 91 ± 3 g/day or 1.03 ± 0.03 g/kg/day, protein group 128 ± 5 g/day or 1.53 ± 0.05 g/kg/day; all *P *<* *0.001). The exchange of 60 g/day protein or 60 g/day maltodextrin supplements for 60 g/day dietary carbohydrates was isocaloric because body weight did not change significantly during the intervention (Teunissen‐Beekman et al. 2012).

**Table 1:** Baseline characteristics

	Maltodextrin group	Protein group	*P*‐value
Gender (m/f)	30/13	25/11	–
Age (year)	55 ± 1	55 ± 1	0.83
BMI (kg/m^2^)	29 ± 0.5	28 ± 0.4	0.10
SBP (mmHg)	143 ± 2	143 ± 2	0.90
DBP (mmHg)	92 ± 1	93 ± 1	0.80

Values are mean ± SEM. Between‐group differences were tested with an independent samples *t*‐test. DBP, diastolic blood pressure; SBP, systolic blood pressure.

### Renal hemodynamic responses

**Figure 1:**
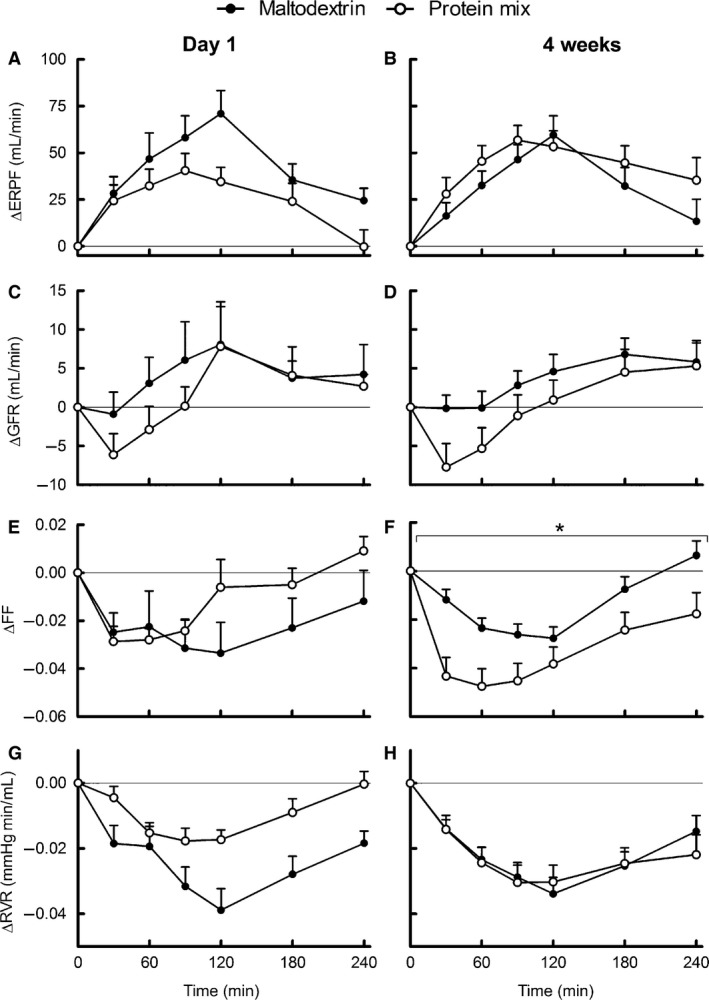
Mean (±SEM) of 4‐h postprandial responses of renal function to breakfasts supplemented with protein or maltodextrin on day 1 of supplementation (left) and after 4 weeks of supplementation (right). Panels are ERPF (A, B), GFR (C, D), FF (E, F), and RVR (G, H). On day 1 ERPF, GFR and FF *n = *25 maltodextrin group, *n = *25 protein group; RVR *n = *24 maltodextrin group, *n = *24 protein group. After 4 weeks ERPF, GFR, FF, and RVR *n = *26 maltodextrin group, *n = *24 protein group. *P < 0.05 for the difference between maltodextrin group (black) and protein group (white) over the whole 4‐h period according to the mixed model. ERPF, effective renal plasma flow; FF, filtration fraction; GFR, glomerular filtration rate; RVR, renal vascular resistance.

Fasting renal hemodynamic values did not differ between groups at baseline and after 4 weeks of supplementation (Table 2). Fasting GFR and ERPF measurements correlated well with each other (Pearson *r* = 0.69). Food intake increased ERPF and ERBF, and decreased FF and RVR (iAUC, *P *≤* *0.05), whereas the iAUC of the postprandial GFR did not differ from zero. Postprandial responses of ERPF, GFR, ERBF, and RVR did not differ between groups on either test day (Fig. 1A–H). Postprandial FF remained significantly higher after the maltodextrin‐supplemented breakfast than after the protein‐supplemented breakfast after 4 weeks of supplementation (Fig. 1F).

**Table 2:** Fasting renal hemodynamics, hormones, and measures of systemic acidity

	Maltodextrin			Protein		
	Day 1	4 weeks	*N*	Day 1	4 weeks	*N*
Body surface area (m^2^)	2.0 ± 0.0		27	2.0 ± 0.0		25
ERPF (mL/min)	440 ± 21	437 ± 20	24	433 ± 17	412 ± 15	24
GFR (mL/min)	137 ± 5	134 ± 5	24	130 ± 5	127 ± 5	24
FF	0.32 ± 0.01	0.31 ± 0.01	24	0.30 ± 0.01	0.32 ± 0.01	24
ERBF (mL/min)	788 ± 36	763 ± 39	23	785 ± 34	710 ± 28	23
RVR (mmHg·min/mL)	0.15 ± 0.01	0.15 ± 0.01	24	0.14 ± 0.01	0.15 ± 0.01	24
Renin (pmol/L)	0.54 ± 0.06^a^	0.52 ± 0.08	25	0.85 ± 0.12^a^	0.75 ± 0.09	25
ACE (U/L)	16 ± 1	16 ± 1	26	15 ± 1	14 ± 1	25
Aldosterone (nmol/L)	0.38 ± 0.02	0.37 ± 0.03	27	0.45 ± 0.07	0.40 ± 0.05	25
pH	7.41 ± 0.00	7.40 ± 0.00	25	7.41 ± 0.00	7.41 ± 0.00	25
HCO_3_ (mmol/L)	24 ± 1	25 ± 1	25	25 ± 1	25 ± 1	25

Values are mean ± SEM. ERPF, effective renal plasma flow; FF, filtration fraction; GFR, glomerular filtration rate; ERBF, effective renal blood flow; RVR, renal vascular resistance.

a *P < *0.05 for between‐group differences. Between‐group differences on day 1 were tested with an independent samples *t*‐test. Between‐group differences after 4 weeks were tested with ANCOVA correcting for the fasting value on day 1.

### Renin, aldosterone, ACE

**Figure 2:**
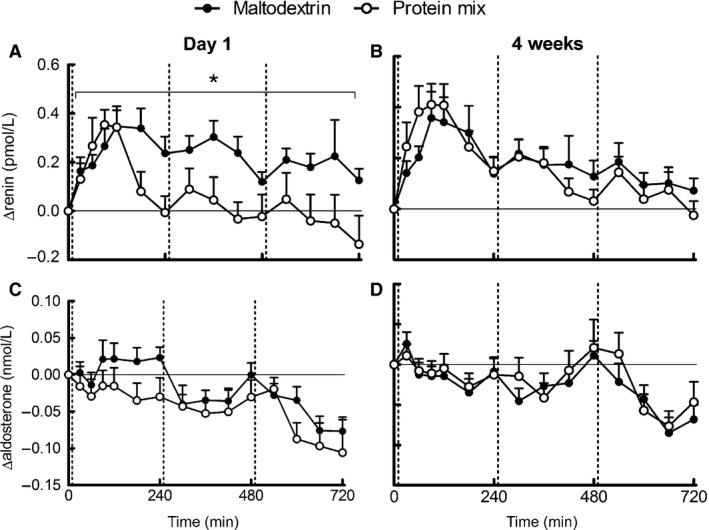
Mean (±SEM) of 12 h postprandial responses of renin‐angiotensin‐aldosterone system to meals supplemented with protein or maltodextrin on day 1 of supplementation (left) and after 4 weeks of supplementation (right). Panels are renin (A, B) and aldosterone (C, D). On day 1 renin *n = *25 maltodextrin group, *n = *25 protein group; aldosterone *n = *27 maltodextrin group, *n = *25 protein group. After 4 weeks renin and aldosterone *n = *27 maltodextrin group, *n = *25 protein group. Vertical lines indicate breakfast, lunch, and dinner. *P < 0.05 for the difference between maltodextrin group (black) and protein group (white) over the whole 12‐h period according to the mixed model.

Baseline renin was significantly lower in the maltodextrin group compared with the protein group (Table 2). Twelve‐hour postprandial renin was increased after the maltodextrin‐supplemented breakfast on day 1 and after both breakfasts at 4 weeks (iAUC, *P *≤* *0.05). Postprandial renin was higher in the maltodextrin group than in the protein group at day 1 of supplementation, but not after 4 weeks (Fig. 2A–B). ACE (data not shown) and aldosterone (Fig. 2C–D) decreased similarly in both groups on both test days (iAUC, *P *≤* *0.05).

### Acid load

**Figure 3:**
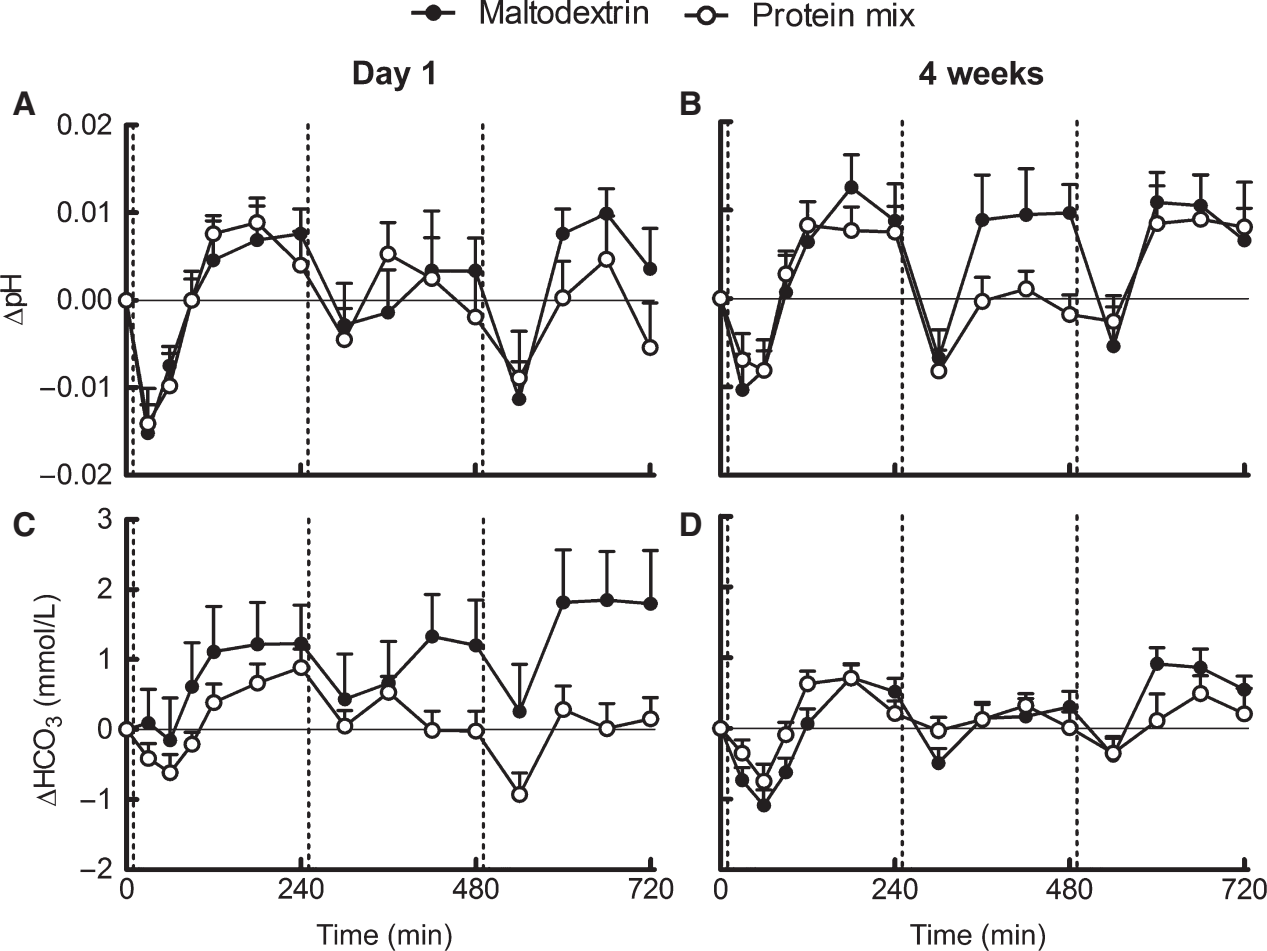
Mean (±SEM) of 12 h postprandial responses of blood pH and bicarbonate to meals supplemented with protein or maltodextrin on day 1 of supplementation (left) and after 4 weeks of supplementation (right). Panels are pH (A, B) and bicarbonate (C, D). On day 1 and after 4 weeks pH and bicarbonate *n = *26 maltodextrin group, *n = *25 protein group. Vertical lines indicate breakfast, lunch, and dinner. Postprandial responses did not differ between the maltodextrin group (black) and protein group (white) according to the mixed model.

**Figure 4:**
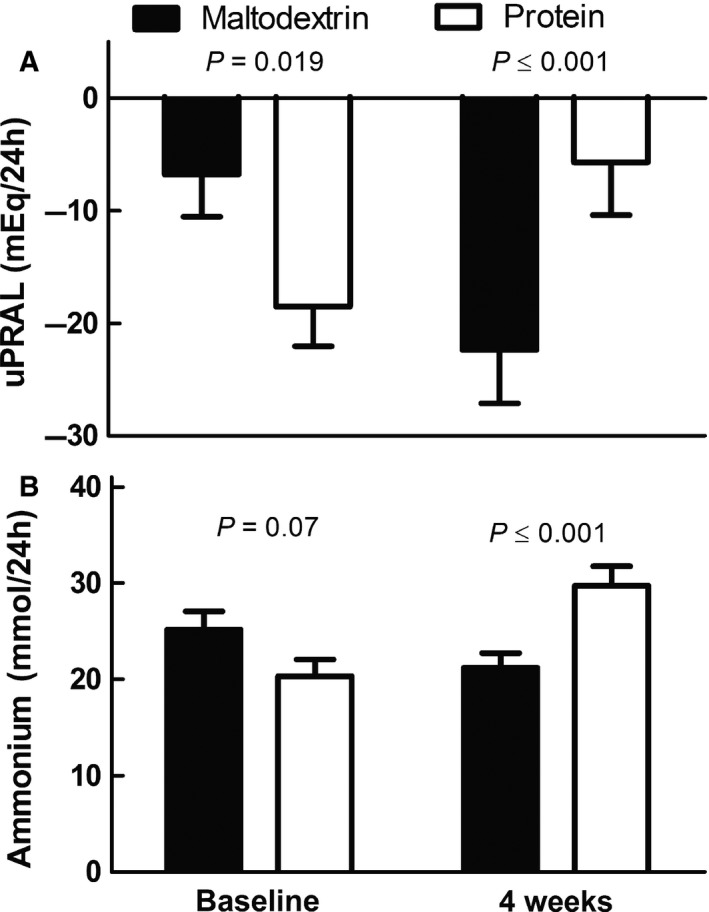
Mean (±SEM) of 24‐h uPRAL (panel A) and ammonium excretion (panel B) at baseline and after 4 weeks of supplementation in the protein group (white) and maltodextrin group (black). For uPRAL: baseline *n* = 41 maltodextrin group, *n* = 35 protein group. After 4 weeks *n* = 38 maltodextrin group, *n* = 35 protein group. For ammonium: baseline *n* = 43 maltodextrin group, *n* = 35 protein group. After 4 weeks *n* = 39 maltodextrin group, *n* = 35 protein group. Between‐group differences on day 0 were tested with an independent samples *t*‐test. Between‐group differences after 4 weeks were tested with ANCOVA correcting for the fasting value on day 0. uPRAL, urinary potential renal acid load.

Fasting and postprandial plasma pH and bicarbonate did not differ between groups. (Fig. 3A–D), and the iAUCs did not significantly differ from zero. Baseline uPRAL (day 0) was significantly lower in the protein group compared with the maltodextrin group, whereas baseline‐corrected uPRAL after 4 weeks was significantly higher in the protein group compared with the maltodextrin group (Fig. 4A). In addition, urinary pH was 0.3 ± 0.1 higher in the maltodextrin group after 4 weeks (*P *=* *0.004), whereas urinary titratable acid did not differ between groups. Ammonium excretion was also significantly higher in the protein group after 4 weeks (Fig. 4B). When only participants with <20% change in 24‐h urinary creatinine were analyzed (maltodextrin group *N* = 27, protein group *N* = 29), baseline uPRAL no longer differed between groups, whereas differences in baseline‐corrected uPRAL and ammonium after 4 weeks sustained.

## Discussion

In contrast to our hypothesis, fasting GFR and other variables of renal hemodynamics did not differ between the protein and maltodextrin group after 4 weeks of supplementation, despite a 52 g/day higher protein intake in the protein group, suggested by 206 mmol/24‐h higher urea excretion in the protein group (Teunissen‐Beekman et al. 2012). Therefore, this study revealed no indication that GFR would be affected by consumption of 60 g protein extra per day for 4 weeks. Increased protein consumption has been reported to increase fasting GFR in energy‐balanced diets (Frank et al. 2009; Juraschek et al. 2013). In one study GFR and FF increased in healthy young men after 7 days on a high‐protein diet (181 g protein/day) versus a normal protein diet (88 g protein/day)(Frank et al. 2009). In the Omniheart trial, 6 weeks on an increased protein diet (+10% of energy from protein) increased fasting GFR compared to diets replacing protein with either carbohydrate or fat in individuals with untreated elevated blood pressure (Juraschek et al. 2013). High‐protein weight‐loss diets also increased fasting GFR (Skov et al. 1999; Tirosh et al. 2013). An explanation why this study did not find an increase in fasting GFR could be because we increased protein intake with protein isolate, whereas others used protein‐rich foods (Skov et al. 1999; Frank et al. 2009; Juraschek et al. 2013; Tirosh et al. 2013). Also, participants in three of these studies had lower baseline GFR (Skov et al. 1999; Juraschek et al. 2013; Tirosh et al. 2013) compared to our participants. Participants in one study had a similar baseline GFR (Frank et al. 2009) as those in our study, however, the contrast in protein intake was higher than in our study (93 g/day vs. 60 g/day) (Frank et al. 2009), and the extra protein consisted mainly of animal protein (Frank et al. 2009), while we used 60% animal protein and 40% plant protein. Plant protein may have a smaller or no effect on GFR. In a crossover trial, consumption of bread containing 80 g/day of wheat gluten versus 93 g/day of carbohydrates for 1 month showed no differences in creatinine clearance in individuals with hyperlipidemia (Jenkins et al. 2001), whereas a crossover study comparing 4 weeks of animal protein consumption with plant protein consumption found higher GFR and ERPF after the animal protein diet in type 1 diabetic patients (Kontessis et al. 1995).

Changes in uPRAL and ammonium excretion showed that, renal acid load was increased in the protein group after 4 weeks, whereas fasting blood pH and bicarbonate did not differ between groups. This suggests that systemic acid‐base balance was not changed by the high‐protein diet. Associations between protein intake and renal acid load have been reported (Remer and Manz 1994), and it has been suggested that increased renal excretion of ammonia and acids might lead to tubulointerstitial injury and decreased kidney function in the long term in patients with diabetic nephropathy (van den Berg et al. 2011). This study found no effects of 4 weeks of increased protein intake on kidney function in our study population with normal kidney function. In addition, albumin excretion was borderline significantly lower in the protein group compared to the maltodextrin group (Teunissen‐Beekman et al. 2012). In agreement with our study, randomized trials have not confirmed disturbances in renal function after high protein diets in individuals with normal kidney function (Bie and Astrup 2015; Campbell and Rains 2015). Six months on a high‐protein (25% of energy from protein) weight‐loss diet was found to increase GFR without adverse changes in albuminuria (Skov et al. 1999). Two years on a low‐carbohydrate high‐protein diet (22% of energy from protein) was found to improve GFR and urinary microalbumin‐to‐creatinine ratios to the same extent as a Mediterranean diet and a low fat diet (19% of energy from protein) (Tirosh et al. 2013). Another 2‐year weight‐loss study found no effects on GFR or albuminuria of a low‐carbohydrate high‐protein diet (Friedman et al. 2012). This suggests that, high‐protein diets do not adversely affect kidney function in individuals with normal kidney function over a period of 2 years, although effects on the longer term still cannot be excluded. In type 2 diabetic patients with early renal disease, 1 year on a moderate protein weight‐loss diet (110 g protein/day) compared with a standard protein diet (97 g protein/day) was not harmful for kidney function (Jesudason et al. 2013). Despite evidence supporting our findings of no adverse effects of increased protein intake on kidney function in individuals with normal renal function or early renal disease, our findings might have been confounded by higher sodium intake in the maltodextrin group (Teunissen‐Beekman et al. 2012). High sodium intake can adversely affect renal function via an interaction with aldosterone and by inducing hyperfiltration (Lambers Heerspink et al. 2012). However, we found no evidence for between‐group differences or increases in either aldosterone or GFR after the intervention in the maltodextrin group (Table 2). Therefore, we do not expect that higher sodium intake in the maltodextrin group confounded our results.

In contrast to our second hypothesis, postprandial responses of GFR and ERPF did not differ after the protein‐ and maltodextrin‐supplemented meals. Also, GFR did not increase significantly after the protein‐supplemented breakfast and only exceeded baseline levels from 90 to 120 min after breakfast. This increase may be too late to be picked up by iAUC analyses. Many studies have reported that protein‐induced increases of postprandial GFR and ERPF (Bosch et al. 1986; Hostetter 1986; Viberti et al. 1987; Simon et al. 1998). Two of these studies did not report AUCs, but found a significant increase in GFR and ERPF by comparing separate time points with baseline measurements (Viberti et al. 1987) or with a control condition (Hostetter 1986). Another potential explanation for the lack of such effect in this study is the relatively low dose of protein consumed. Moreover, compliance was not complete, as estimated for protein intake from 24‐h UNN excretion. Studies that reported increases in GFR gave 70–90 g of protein (Bosch et al. 1986; Viberti et al. 1987; Kontessis et al. 1990; Simon et al. 1998), whereas the protein content of the protein‐supplemented breakfast in our study was 43 g. Differences in protein sources tested may also lead to different outcomes. Egg‐white protein, cheese, tofu and soy were found to have no acute effects on GFR and ERPF (Kontessis et al. 1990; Nakamura et al. 1990), whereas meat or tuna increased GFR (Bosch et al. 1986; Hostetter 1986; Viberti et al. 1987; Kontessis et al. 1990; Nakamura et al. 1990; Simon et al. 1998). The sulfur‐containing amino acids methionine and cysteine could play a role in the increased GFR after meat and tuna intake (Brosnan and Brosnan 2006). However, the protein mix in this study also contained a considerable amount of methionine and cysteine, which is reflected in increased urinary sulfate excretion in the protein group during the test days (data not shown) and after 4 weeks of supplementation (Teunissen‐Beekman et al. 2012). Finally, the high baseline GFR in our study participants may point toward an exhausted nephron reserve, which results in the inability to increase GFR any further. However, we found no relationship between baseline GFR and the maximal GFR response after consumption of a high‐protein breakfast (Pearson *r* = 0.02) or maltodextrin breakfast (Pearson *r* = −0.35). Meal consumption increased ERPF in our study, with no difference between meal types. This increase in ERPF was accompanied by a decrease in RVR and parallels the decrease in blood pressure and total peripheral resistance reported earlier (Teunissen‐Beekman et al. 2013). Thus, increased regional blood flow in the kidneys partly contributed to the postprandial decrease in blood pressure and total peripheral resistance.

On the first day of supplementation, but not after 4 weeks, renin increased to a higher level after the maltodextrin‐supplemented breakfast. The meal‐induced increase in renin could be a homeostatic response to the meal‐induced decrease in blood pressure, which was previously reported (Teunissen‐Beekman et al. 2013). This idea is strengthened by the finding that renin was significantly higher in the maltodextrin group on day 1, when blood pressure was significantly lower (Teunissen‐Beekman et al. 2013), whereas postprandial responses of renin and blood pressure did not differ after 4 weeks (Teunissen‐Beekman et al. 2013). Higher postprandial renin levels could be a result of the higher insulin response (Perlstein et al. 2007) in the maltodextrin group (Teunissen‐Beekman et al. 2013). However, postprandial renin no longer differed between groups after 4 weeks of supplementation despite higher postprandial insulin in the maltodextrin group, which makes this explanation less likely. In addition, we found no between‐group differences in ERBF response on either test day, which contrasts with findings on insulin‐induced renal vasodilation (Perlstein et al. 2007). Aldosterone decreased below baseline during the day but showed no clear meal‐induced responses. This postprandial change could reflect the circadian rhythm of aldosterone, which peaks at 0600 and then lowers during the day until 00:00 (Hurwitz et al. 2004). Consumption of beef containing 1 g/kg bodyweight of protein was found to increase aldosterone after two hours (Krishna et al. 1988), but our protein dose was much smaller.

A limitation of our study was that our findings could be a result of increased protein intake as well as a result of decreased carbohydrate intake. This limitation was inevitable because increased intake of one macronutrient will always result in decreased intake of at least one of the other macronutrients under isocaloric conditions (Altorf‐van der Kuil et al. 2010; Teunissen‐Beekman et al. 2012).

In conclusion, this study shows that a moderate increase in protein intake to ≈25% of energy by replacing 60 g of maltodextrin per day by 60 g of a protein isolate mixture (20% pea protein, 20% soy protein, 40% milk protein, 40% egg‐white protein) per day for 4 weeks increases the systemic acid load compared to maltodextrin intake, with no evidence for any effects on kidney function in healthy obese individuals with mildly elevated blood pressure and normal kidney function.

## Conflict of Interest

None declared.
